# Prevalence of Fractures and Diagnostic Accuracy of Emergency X-ray in Older Adults Sustaining a Low-Energy Fall: A Retrospective Study

**DOI:** 10.3390/jcm9010097

**Published:** 2019-12-30

**Authors:** Alina Lampart, Isabelle Arnold, Nina Mäder, Sandra Niedermeier, Armin Escher, Robert Stahl, Christoph Trumm, Christian Kammerlander, Wolfgang Böcker, Christian H. Nickel, Roland Bingisser, Vera Pedersen

**Affiliations:** 1 Department of Emergency Medicine, University Hospital Basel, Petersgraben 2, 4031 Basel, Switzerland; alina.lampart@usb.ch (A.L.); isabelle.arnold@unibas.ch (I.A.); christian.nickel@usb.ch (C.H.N.); roland.bingisser@usb.ch (R.B.); 2 Department for General, Trauma and Reconstructive Surgery, Ludwig Maximilian University Munich, Marchioninstr. 15, 81377 Munich, Germany; sandra.niedermeier@med.uni-muenchen.de (S.N.); christian.kammerlander@med.uni-muenchen.de (C.K.); wolfgang.boecker@med.uni-muenchen.de (W.B.); 3 Department of Radiology, University Hospital Basel, Petersgraben 2, 4031 Basel, Switzerland; armin.escher@usb.ch; 4 Institute of Diagnostic and Interventional Neuroradiology, Ludwig Maximilian University Munich, Marchioninstr. 15, 81377 Munich, Germany; robert.stahl@med.uni-muenchen.de (R.S.); christoph.trumm@med.uni-muenchen.de (C.T.)

**Keywords:** low-energy fall, older adult, computed tomography, fracture, X-ray

## Abstract

Background: Plain radiography (XR) series are standard of care for detection of fall-related fractures in older patients with low-energy falls (LEF) in the emergency department (ED). We have investigated the prevalence of fractures and diagnostic accuracy of XR imaging in the ED. Methods: 2839 patients with LEF, who were presented to two urban level I trauma centers in 2016 and received XR and computed tomography (CT), were consecutively included in this retrospective cohort study. The primary endpoint was the prevalence of fractures of the vertebral column, rib cage, pelvic ring, and proximal long bones. Secondary endpoints were diagnostic accuracy of XR for fracture detection with CT as reference standard and cumulative radiation doses applied. Results: Median age was 82 years (range 65–105) with 64.1% female patients. Results revealed that 585/2839 (20.6%) patients sustained fractures and 452/2839 (15.9%) patients received subsequent XR and CT examinations of single body regions. Cross-tabulation analysis revealed sensitivity of XR of 49.7%, a positive likelihood ratio of 27.6, and negative likelihood ratio of 0.5. Conclusions: XR is of moderate diagnostic accuracy for ruling-out fractures of the spine, pelvic ring, and rib cage in older patients with LEF. Prospective validations are required to investigate the overall risk–benefit of direct CT imaging strategies, considering the trade-off between diagnostic safety, health care costs, and radiation exposure.

## 1. Introduction

Low-energy falls (LEF) occur in one-third of adults over the age of 65 each year, and are a leading cause of death in developed nations [1]. The emergency department (ED) visit rates for LEF among older adults are increasing [2]. In the United States of America 20%–30% of older people who have fallen suffer moderate to severe injuries, such as bruises, hip fractures or head trauma [3]. LEF are associated with significant morbidity and mortality that appear to increase with age [3,4]. Trauma registry analysis emphasizes that LEF are the predominant trauma mechanism of older individuals leading to injury severities similar to high-energy mechanisms in younger patients [5].

Assessment and diagnostic evaluation of these patients are difficult, and they are jeopardized by a systemic underestimation of the trauma mechanism, resulting in potentially severe or even life-threatening injuries and often complicated medical or neurological conditions [6,7,8,9].

The majority of older adults suffering from LEF presented themselves to the ED as walk-in patients or with emergency medical services without previous trauma-team activation. Diagnosis of skeletal injuries is predominantly performed by plain radiography (XR). However, XR might miss a substantial portion of fractures of the rib cage and pelvic ring [10,11,12,13]. Evaluation of the vertebral column after LEF is frequently limited to computed tomography (CT) of the cervical spine [14]. Supplementary imaging of the thoracolumbar spine often depends on clinical presentation and clinical experience of the treating physician. Moreover, history and physical examination findings are generally inaccurate to rule-in or rule-out fractures of the thoracolumbar spine [15], and the current guidelines [16,17] cannot be readily applied to older adults with LEF.

The objectives of this study were to analyze the prevalence of fractures of the axial skeleton (vertebral column, sternum, rib cage), the pelvic ring, and the proximal long bones, and to measure the diagnostic accuracy of XR and the cumulative radiation doses applied to older adults after LEF with radiological imaging.

## 2. Materials and Methods

### 2.1. Study Design and Setting

This bicentric, binational retrospective study was carried out in two university tertiary care hospitals in Switzerland (University Hospital Basel) and Germany (University Hospital of Ludwig Maximilian University Munich) using electronic health records (EHR). The study is in accordance with the Declaration of Helsinki and was conducted using STROBE (Strengthening the Reporting of Observational Studies in Epidemiology) guidelines. Ethics approval was obtained from local ethics committees (EKNZ 2017-01078, EK LMU 17-217).

### 2.2. Study Population

The study population includes individuals ≥65 years of age who presented to one of the two EDs from 1 January 2016 to 31 December 2016 and received a CT examination of the axial skeleton, pelvic ring or proximal long bones within 48 h of the index visit. All the individuals suffered from a documented LEF, including fall from standing height (W00, W01, W03, W04, and W18), fall out of bed/from chair/wheel-chair or other low level furniture (W05–W08) or fall from low level (W10, if ≤1 m) (according to International Statistical Classification of Diseases and Related Health Problems 10th Revision (ICD-10) [18]) in the last 7 days before ED presentation.

Exclusion criteria were: initial presentation via resuscitation room, referral from general practitioners or other hospitals with preceding imaging, presentation via fast track process, delayed presentation (≥8 days after the fall), re-presentation due to the same incident, CT examinations solely distal from knee or elbow, and XR examination following diagnostic CT examination.

### 2.3. Data Collection

Radiology information systems (RISs) in both study centers were screened for patients aged 65 and older receiving a CT examination within 48 h of their index visit. EHR of all retrieved cases were manually screened for documented LEF. Baseline demographics, final injury diagnosis (from the final discharge report), number of fractures per imaging modality (from the final board certified radiologist’s report), number, and modality of XR and CT studies were extracted from the EHR by two trained observers (A.L., I.A., N.M., and V.P.). Disagreements or equivocalness was decided upon by a third observer (A.L. and V.P.) uninvolved in the initial extraction. Screening and chart review abstraction were conducted in accordance with the recommendations for medical chart review [19,20], which were fulfilled for 11 of 12 guidelines (abstractors were not blinded to the hypothesis). Interrater agreement for inclusion criteria was determined using corresponding 95% confidence interval. Double data entry was performed in a Microsoft Access 2010/2016 database (Microsoft, Redmond, Washington, DC, USA).

Detailed description of calculation of injury severity score (ISS) and estimation of cumulative radiation doses are provided in the online-only Supplementary Methods.

### 2.4. Key Outcome Measures

The primary endpoint of the study was to assess the prevalence of fractures in older patients with LEF. Secondary endpoints were the measurements of diagnostic accuracy of XR and cumulative radiation doses. Measures of diagnostic accuracy were: sensitivity and specificity, positive (PPV) and negative predictive values (NPV), positive (LR^+^) and negative likelihood-ratios (LR^−^), and the accuracy (diagnostic effectiveness) with CT set as a reference standard [21]. Prevalence of missed fractures in XR of the axial skeleton, pelvic ring, humerus, femur, and others (clavicle, scapula), and performance of CT examination following XR examination of the same body region were assessed.

### 2.5. Statistics

For descriptive statistics, arithmetic means or medians with ranges or interquartile ranges (IQR) were used as appropriate. For comparisons in categorical values, Pearson’s Chi-squared test and Fisher’s exact test were used; for comparisons in scaled or normally distributed values, the *t*-test or false discovery rate (FDR) correction according to Benjamini-Hochberg was performed as appropriate. A binary logistic regression model was calculated to identify risk factors (age class, gender, disposition, in-hospital mortality, trauma mechanism, ISS, and study center) for performance of CT examination after XR examination of the same body region. A *p*-value <0.05 was considered significant. Statistical analyses were performed using SPSS Statistics 22 and R version 3.5.2.

## 3. Results

### 3.1. Baseline Data

**Figure 1:**
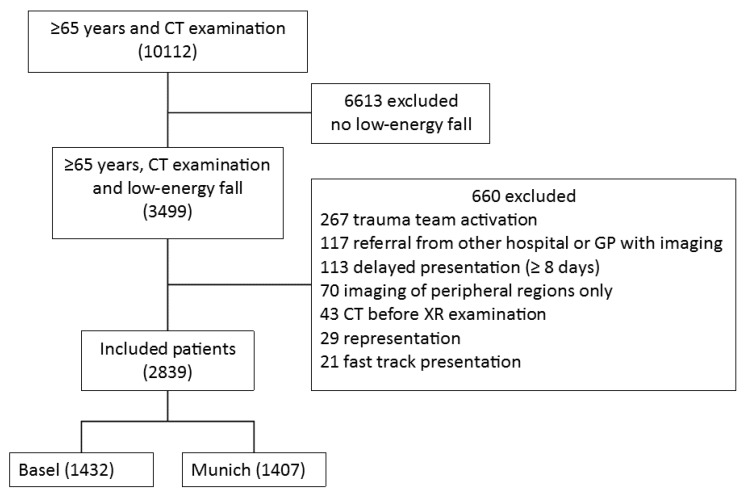
Inclusion and exclusion flow diagram of patient selection from 1 January 2016 to 31 December 2016 in Basel and Munich, receiving computed tomography (CT) examination of the head, spine, chest, pelvic ring or proximal long bones during emergency department (ED) presentation or within 48 h. GP = general practitioner, XR = plain radiography.

We identified 10,112 cases that presented to the ED of the two study centers between January 2016 and December 2016 and received a CT examination. In 3499 (34.6%) cases, LEF were related to the index presentation. Finally, 2839 cases were included in both centers (Figure 1) with a median age of 82 (range 65–105), of which 1821 (64.1%) were female (Table 1). Detailed characteristics and between centers comparisons are reported in the online-only Supplementary Results. Interrater agreement for patient inclusion was 94.3% (95% CI: 93.1–95.5).

### 3.2. Imaging Patterns

Three patterns of imaging were identified for each investigated body region: (1) “XR examination only”; (2) “CT examination only”; and (3) “XR before CT examination”. In 452/2839 (16%) patients “XR before CT examination” was performed in at least one of the body regions of interest, while 464/2839 (16.3%) patients received solely head CT. The remaining 1923/2839 (67.7%) patients received XR or CT or both examinations in different body regions of interest.

Results of regression analysis are shown in Table 2. For performing “XR examination only” or “CT examination only” of a respective body, patients from 65 to 74 years of age had significantly increased odds. Female patients were more likely to receive “XR before CT examination”. Patients with a fall from standing position had significantly increased odds of CT examination following XR examination of a distinct body region. Patients discharged from the ED were more likely to have received “XR examination only” or “CT examination only”.

**Figure 2:**
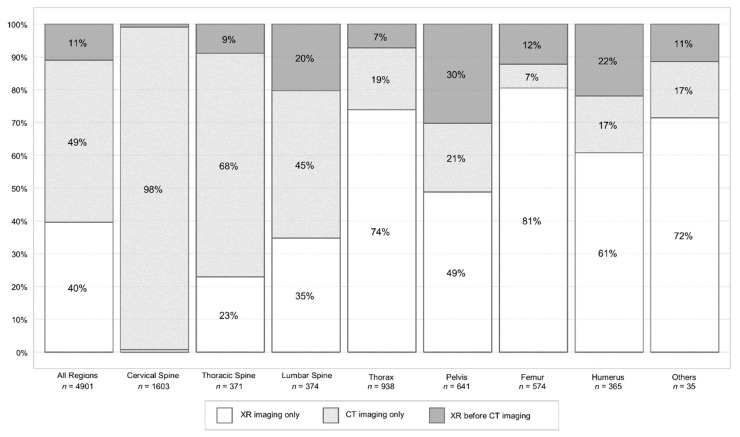
Proportion (%) of imaging work-flows “XR imaging only”, “CT imaging only”, and “XR before CT imaging” (*n* = 4901 imaging processes) of the cervical spine, thoracic spine, lumbar spine, rib cage, pelvic ring, proximal femur and humerus, and other regions (clavicle, scapula, sternum, and coccyx). CT = computed tomography, XR = plain radiography.

Altogether, 4901 single imaging procedures (in 2375/2839 cases) of either identified pattern were found. Of 4901 procedures, 540 (11%) were identified as “XR before CT examination” (Figure 2). The highest incidences for sequentially imaging were observed in the pelvic ring with 194/641 (30%), the proximal humerus with 80/365 (22%), and the lumbar spine with 76/374 (20%) of the examinations (Figure 2). The cervical spine was imaged by “CT examination only” in 1577/1603 (98%) examinations. Center specific imaging approaches are reported in Table S1.

### 3.3. Prevalence of Fractures and Diagnostic Accuracy Measurements

Fractures were detected in 585/2839 (20.6%) patients in the investigated skeletal regions by XR, CT or both. Fracture prevalence was calculated as follows: cervical spine 39/2839 (1.4%), thoracic spine 62/2839 (2.2%), lumbar spine 71/2839 (2.5%), rib cage 86/2839 (3.0%), pelvic ring 152/2839 (5.4%), humerus 112/2839 (3.9%), femur 112/2839 (3.9%), and others 18/2839 (0.6%).

**Figure 3:**
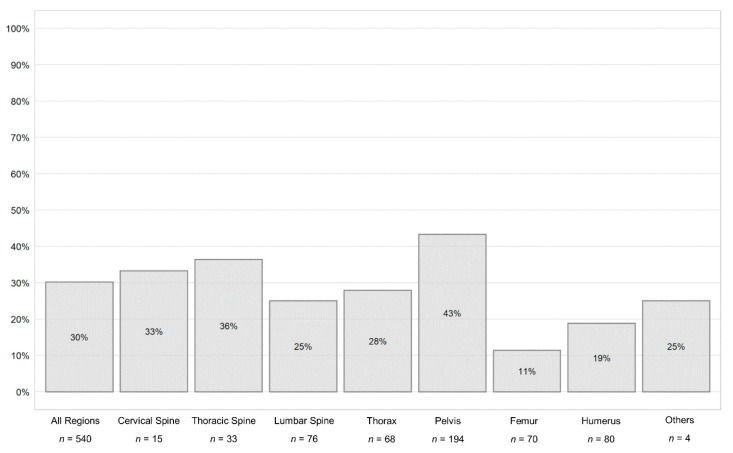
Cumulative percentage of diagnosis that would have been inaccurate if CT had not been performed in “XR before CT examination” (*n* = 540 imaging processes) of the cervical spine, thoracic spine, lumbar spine, rib cage, pelvic ring, proximal femur and humerus, and other regions (clavicle, scapula, sternum, and coccyx).

Cross-tabulation was performed for 540 “XR before CT examination” procedures. Measurements of diagnostic accuracy of XR to detect fractures with CT set as reference standard were calculated for the different body regions (Table 3). Overall, sensitivity of XR to detect fractures was 49.7% (95% CI: 44.0–55.3) and specificity was 98.2% (95% CI: 95.5–99.5). The PPV was 97.5% (95% CI: 93.7–99.1), NPV was 58.0% (95% CI: 55.3–60.7), LR^+^ was 27.6 (95% CI: 10.5–74.0), and LR^−^ was 0.5 (95% CI: 0.5–0.6) (Table 3). Figure 3 illustrates the cumulative percentage of diagnosis that would have been inaccurate if CT had not been performed. A detailed summary of false positive, false negative, true negative, and true positive diagnosis of XR are reported in Table S2.

### 3.4. Effective Dose Estimation

Effective dose estimations were accomplished in 2484 cases (87.5%). The highest effective doses of median 9.14 mSv (range 0.14–46.4 mSv, IQR: 5.94–15.6 mSv) were administered in cases with “CT examination only”. This was significantly higher than in cases with “XR before CT examination” with a median effective dose of 5.50 mSv (range 0.03–49.6 mSv, IQR: 3.17–9.27; *p* < 0.001 post-hoc FDR according to Benjamini–Hochberg). Detailed dose estimations in the investigated skeletal regions and comparison to previously published data are reported in Tables S3 and S4.

## 4. Discussion

Low-energy falls of older adults are associated with significant morbidity and mortality [3,4] despite the low-impact trauma mechanism. Since clinical assessment and diagnostic evaluation of these patients are difficult, the systemic underestimation of their fall-related, potentially life threatening injuries exposes them to the risk of undiagnosed injuries or unfavorable outcomes [6,7,8,9].

Standard of care diagnosis of skeletal injuries relies on plain XR, although XR might miss a substantial portion of fractures of the rib cage and pelvic ring [10,11,12,13]. Evaluation of the vertebral column is frequently limited to imaging of the cervical spine [14], whereas supplementary imaging of the thoracolumbar spine often depends on clinical presentation and clinical experience of the treating physician, which might be submitted to diagnostic inaccuracy [15].

The main result of this study, representing a subset of a large patient cohort recently published [22], is that one out of five older adults with LEF, recorded at the ED, has suffered from fractures of the axial skeleton, pelvic ring or proximal long bones, as diagnosed by XR and CT examinations. Fractures of the pelvic ring, proximal femur, and humerus show the highest prevalence in this cohort.

Overall, our observation demonstrates that in one out of five XR examinations, sequel CT examinations of the same body region were requested for diagnostic assertion. For sequel examinations cross-tabulation analysis demonstrated that fracture detection by XR is on the one hand specific, but, on the other hand, a negative XR does not safely rule-out fractures in the investigated body region. In our study, XR showed the lowest diagnostic accuracy for fracture detection in the pelvic ring, cervical and thoracic spine, and rib cage.

The prevalence of 5.4% for pelvic ring fractures in our cohort is slightly lower than the previously described 7.2% in a large trauma registry analysis [23]. This might be explained by a potential preselection of more severely injured LEF patients, who are eligible for trauma registry inclusion. We found that 43% of XR examinations followed by CT examination missed one or more fractures of the pelvic ring. Therefore, we suspect the real prevalence for pelvic ring fractures to be higher in our cohort. The specificity of XR for fracture diagnosis was 98.6% and the LR^+^ was 22.4, suggesting that patients with an XR-detected fracture of the pelvic ring did indeed have a pelvic ring fracture. On the other side, a LR^−^ of 0.6 indicates that a negative XR does not safely rule-out a fracture of the pelvic ring. These findings are confirmed by few other studies on the targeted population. Heikal et al. found 58% of hip and pelvic ring fractures were missed in XR [11]. Thomas et al. showed that from 199 negative XR of the pelvic ring, 55% of the fractures of the pelvic ring and the proximal femur were missed [13]. Another study with consecutive imaging of the pelvic ring yielded a LR^−^ of 0.89 and 0.27 for detection of sacral fractures and pubic bone fractures, respectively [12]. In these studies, the majority of undetected fractures were located in the dorsal pelvic ring and sacrum. This defines fragility fractures of the pelvis Type II to IV [24], which might require surgical therapy when unstable or provoke prolonged, pain-induced immobilization of the patient [24,25,26]; this, in our opinion, demands diagnostic assurance.

Rib fractures were XR and CT detected in 3.0% of the patients in our cohort. Of these cases with rib fractures, nearly 12% had fractures of more than three ribs, serial and/or bilateral, which were not detected by X-ray. The prevalence was remarkably higher—by 29%—in a recent study, when only patients with LEF and suspected injuries of the rib cage were included for radiological examinations [10]. Furthermore, in our analysis the measurements of diagnostic accuracy for chest XR to detect rib fractures revealed low sensitivity (22.7%) and likelihood ratios (LR^+^ 5.3, LR^−^ 0.8), demonstrating that application of chest XR examination does not safely rule-in or rule-out fractures of the rib cage. This observation is confirmed by the retrospective cohort study of Singleton et al. on 330 non-consecutive older patients with LEF and trauma room presentation, where chest CT followed chest XR [10]. They showed a sensitivity of 42% of chest XR and a LR^−^ of 0.6 of XR to rule-out rib fractures. However, in their study the diagnosis of rib fractures did not result in differences of the length of hospital stay, intensive care unit (ICU) admission rate or in-hospital mortality, which was remarkably higher—by 10.3%—in patients with CT-detected rib fractures [10]. Further studies on blunt trauma patients of every age have demonstrated that chest XR misses rib fractures and relevant intrathoracic injuries in blunt trauma patients of every age [27,28,29]. The proportion of undiagnosed rib fractures in chest XR ranges from 45% [29] to 74.5% (median three additional fractures in CT) [27]. Intrathoracic injuries have been CT-identified in 26% of the cases [28], leading to changes in clinical management in 8% [28], respectively 34.5% [27] of the cases. Additionally, it has been demonstrated that mortality increases by 19% with each additional rib fracture in older patients with blunt trauma [30]. Bearing this in mind, it appears to be clinically relevant to know whether one or two ribs are fractured or three and more, defining a multiple or serial rib fracture. The latter represents a severe blunt chest trauma with different prognostication, requirement for more aggressive pain management and functional therapy and the potential for surgical intervention in case of more part fractures or fracture displacement.

We found the prevalence for cervical spine fractures to be lower by 1.4%. CT examination of the cervical spine has been performed in 98% of cases when imaging of the cervical spine is required according to clinical decision rules [31,32] and current guidelines [14,16]. Importantly, when XR of the cervical spine was performed prior to CT examination on physicians’ decisions in 15 cases, XR missed one of three fractures. Due to the inferior diagnostic capability of XR to detect fractures of the cervical spine, first-line CT examination should be the emergency imaging modality of choice to detect or rule-out fractures in this vertebral column region.

Prevalence for fractures of the thoracic and lumbar spine is 2.2% and 2.5%, respectively. This is consistent with a multi-center trauma registry analysis showing prevalence of 1.8% for thoracolumbar spine fractures in older individuals with LEF and multi-level injuries in 9.6% of patients with vertebral fractures [33]. In our observation, XR is specific for fractures of the thoracic and lumbar spine, but cross-tabulation for sensitivity and calculations of the LR^−^ (thoracic spine: 0.6; lumbar spine: 0.4) demonstrate that XR is not capable to safely rule-out fractures of these regions. Our findings are supported by a recently published meta-analysis, which demonstrates a pooled LR^−^ of 0.43 for XR to detect fractures of the thoracolumbar spine in adults with blunt, high- and low-energy injury mechanisms [15]. Current guideline recommendations are inconsistent as regards imaging modalities. The Eastern Association for the Surgery of Trauma (EAST) practices CT examination as the screening modality of choice when imaging is deemed necessary in blunt trauma patients [17]. The recent National Institute for Health and Care Excellence (NICE)-guidelines recommend performing XR as the first-line investigation for individuals with suspected spinal column injury without abnormal neurological signs or symptoms in the thoracic or lumbosacral regions, and only perform CT when the XR is abnormal in this region [16]. Imaging approaches have to allow rapid and effective clinical decision-making and care [34]. To date, neither guidelines nor available evidence on imaging recommendations for blunt thoracolumbar injuries are satisfactory in quantity and quality, even less so in the older population [15]. Our data now add some new information concerning the older population and low-energy trauma based on a large cohort. However, patient-oriented benefit of a first-line CT examination and the clinical relevance of additionally detected fractures by CT remains to be defined.

With regard to diagnostic accuracy, reformatted thoracolumbar spine CT showed higher accuracy than the chest–abdomen–pelvis CT [15], with an unknown impact on clinical management. However, in this meta-analysis, the pooled LR^+^ was 81.1 (95% CI: 14.1–467.9) and the LR^−^ was 0.04 (95% CI: 0.02–0.08) for diagnosing thoracolumbar spine fractures with the chest–abdomen–pelvis CT. Taking into account these observations and our findings, prospective studies comparing first-line chest–abdomen–pelvis CT examinations with sequential scanning (XR before CT) should be performed in order to tackle the unsolved issue of the moderate diagnostic accuracy of XR in older patients. The improved accuracy of fracture detection using CT seems obvious, but patient-oriented outcomes, such as the number of interventions, the duration of immobility, and the incidence of institutionalization, are to be explored. In our opinion, CT examination should be considered when clinical suspicion is high for injuries of the axial skeleton or pelvic ring in more than one region, physical examination is inconclusive, and further CT examinations such as head and cervical spine CT are necessary in older patients with LEF.

Certainly, there is a trade-off between cost and radiation doses on the one hand, and diagnostic accuracy on the other. Our study demonstrated that the highest effective radiation doses were applied when only CT examinations were performed. This included trunk and whole body contrast enhanced CT examinations. Radiation doses in our study were less, as compared to an all-age major trauma population, which receives an estimated radiation dose of 20.9 mSv by whole body CT examinations [35]. Effective dose estimations of single body regions demonstrated that the highest doses were applied during CT examination of the thoracic and lumbar spine (see Tables S3 and S4). These findings are supported by a previous study, investigating the effective doses of CT scans performed for various musculoskeletal applications [36]. However, lifetime cancer risk from CT examination is the highest for a CT scan of the torso in females, 60 to 69 years of age, with lifetime cancer incidence of 3.6 per 10,000 individuals [37]. This is estimated as an overall “low” risk [38]. With increasing age, the risk constantly declines to “minimal” beyond the age of 90 [37,38].

Our study has several strengths, including a large consecutive sample from two typical European urban tertiary care centers, as well as rigorous chart review abstraction for inclusion criteria and key measurements. Nevertheless, the study is limited by its retrospective design and by initial patient selection. The first limitation is that our observation might underestimate the prevalence of fractures, since most patients and regions were only examined by XR. Secondly, the initial patient selection represents a potential risk for selection bias. However, this inclusion strategy did not miss any patient with the gold standard examination CT, which allows calculation of diagnostic accuracy measurements. The third limitation is that the clinical relevance of our findings remains unknown due to the retrospective approach. We analyzed the rate of surgical treatment by mapping patients into different subgroups of imaging work-up. Based on a logistic regression, we found no differences in the rate of intervention comparing the three groups (XR only, CT only, or XR before CT). From a surgeon’s point of view, only accurate fracture diagnosis allows the evaluation of fracture stability and prognostication. Furthermore, decisions on interventions, communication with patients, and detection of potentially underlying osteoporosis are all based on a sound diagnosis [39]. Our study adds some insight into the diagnostic shortcomings of XR of the axial skeleton in the setting of emergency imaging of older adults after LEF and may facilitate argumentation for CT imaging in case of ambiguous XR findings.

## 5. Conclusions

In conclusion, our study demonstrates that one out of five older patients with LEF has sustained osseous injuries of the axial skeleton, the pelvic ring or the proximal long bones. Standard of care XR has moderate diagnostic accuracy in detecting fractures of the spine, pelvic ring, and rib cage. Supposing that accurate fracture diagnosis is favored in these patients as in any other age group, our findings warrant a higher radiation exposure applied by CT examinations in a first-line imaging approach. However, in order to assess patient-centered clinical outcomes [40] of different imaging strategies, as well as the potential trade-offs between resources, radiation, and fracture detection more broadly, prospective multicenter randomized clinical trials are needed. Subsequent results would help to determine preexisting conditions and risk factors in these patients (e.g., multimorbidity, dementia, manifested osteoporosis or polypharmacy), to elaborate and develop clinical decision rules adjusted to and appropriate for the older population. 

## Figures and Tables

**Table 1:** Baseline characteristics of 2839 older adult patients presenting with low-energy falls from 1 January 2016 to 31 December 2016 and characteristics of patients (*n* = 452) that received “XR before CT imaging”.

Characteristics	Total (*n* = 2839)	Basel (*n* = 1432)	Munich (*n* = 1407)	Patients with XR before CT (*n* = 452)
Age (median, IQR)	82 (71–95)	82 (71–95)	81 (80–94)	83 (82–84) ^b^
65–74 (%)	607 (21.4)	310 (21.6)	297 (21.1)	84 (18.6) *
75–84 (%)	1133 (44.1)	541 (42.9)	592 (45.4)	166 (36.7)
>85 (%)	1099 (34.5)	581 (35.5)	518 (33.5)	202 (44.7)
Female (%)	1821 (64.1)	915 (63.9)	906 (64.4)	342 (75.7) ***
In-hospital mortality (%)	62 (3.3)	26 (2.8)	36 (3.7)	13 (2.9)
Hospital admission (%)	1879 (66.2)	916 (64)	963 (68.4) ^a^	391 (86.5) ***
Trauma mechanism				
Fall from standing (%)	2451 (86.3)	1233 (86.1)	1218 (86.6)	397 (87.8) **
Fall from low furniture (%)	279 (9.8)	144 (10.1)	135 (9.6)	33 (7.3)
Fall <1 m (%)	109 (3.9)	55 (3.8)	54 (3.8)	22 (4.9)
ISS (median, 95% CI)	3 (2–3)	3 (3–4)	3 (3–4)	5 (5–5) ^b^
Non-injurious fall (%)	377 (13.3)	194 (13.5)	183 (13.0)	28 (6.2)

If not otherwise stated, data are reported as number of patients (%). ISS = injury severity score; IQR = interquartile range. Non-injurious fall: ISS = 0. ^a^ *p* < 0.05 between centers (Fisher’s exact test); * *p* < 0.05/** *p* < 0.001 between patients with XR before CT and patients with XR or CT only (Pearson Chi-squared test); *** *p* < 0.001 between patients with XR before CT and patients with XR or CT only (Fisher’s exact test); ^b^ *p* < 0.05 between patients with XR before CT and patients with XR or CT only (*t*-test).

**Table 2:** Binary logistic regression of performing XR or CT separate or XR before CT examination.

Imaging Patterns		XR or CT			XR before CT		
Variable	Level	OR	95% CI	*p*-Value	OR	95% CI	*p*-Value
Age (years)	65–74	119.2	51.3–277.4	<0.001	0.01	0.004–0.2	<0.001
	75–84	1.1	0.4–3.1	0.9	0.9	0.3–2.7	0.9
	≥85	Ref			Ref		
Gender	Female	0.5	0.4–0.8	0.001	1.9	1.3–2.8	0.001
	Male	Ref			Ref		
Trauma mechanism	Fall from standing	0.001	0.0–0.002	<0.001	1053.4	427.0–2599.0	<0.001
	Fall from low furniture	0.7	0.2–2.1	0.5	1.5	0.5–4.9	0.5
	Fall <1 m	Ref			Ref		
Disposition	discharge	3.5	2.3–5.5	<0.001	0.3	0.2–0.4	<0.001
	admission	Ref			Ref		
Mortality	In-hospital	0.8	0.3–2.4	0.7	1.2	0.4–3.6	0.7
	survived	Ref			Ref		
ISS	<10	1.5	0.7–3.4	0.3	0.6	0.3–1.5	0.3
	10–15	1.2	0.5–3.3	0.7	0.8	0.3–2.2	0.7
	>15	Ref			Ref		
Center	Basel	0.8	0.6–1.2	0.2	1.2	0.9–1.8	0.2
	Munich	Ref			Ref		

CT = computed tomography, ISS = injury severity score, OR = odds ratio, XR = plain radiography, 95% CI = 95% confidence interval.

**Table 3:** Summary of measurements of diagnostic accuracy (95% CI) of XR for fracture detection according to computed tomography as reference standard.

Region	Sensitivity (%)	Specificity (%)	PPV (%)	NPV (%)	LR^+^	LR^−^	Accuracy (%)
Cervical spine (*n* = 15)	16.7 (0.4–64.1)	100 (66.4–100)	100 (n.a.)	64.3 (55.7–72.0)	n.a.	0.8 (0.6–1.2)	66.7 (38.4–88.2)
Thoracic spine (*n* = 33)	40.0 (19.1–64.0)	100 (75.3–100)	100 (n.a.)	52.0 (43.1–60.8)	n.a.	0.6 (0.4–0.9)	63.6 (45.1–79.6)
Lumbar spine (*n* = 76)	57.8 (42.2–72.3)	100 (88.8–100)	100 (n.a.)	62.0 (53.7–69.7)	n.a.	0.4 (0.3–0.6)	75.0 (63.7–84.2)
Chest (*n* = 68)	22.7 (7.8–45.4)	95.7 (85.2–99.5)	71.4 (34.5–92.2)	72.1 (67.2–76.6)	5.2 (1.1–24.9)	0.8 (0.6–1.0)	72.1 (59.9–82.3)
Pelvis (*n* = 194)	31.4 (23.3–40.5)	98.6 (92.6–99.9)	97.4 (84.2–99.6)	46.5 (43.4–49.5)	22.9 (3.2–163.5)	0.7 (0.6–0.8)	56.7 (49.4–63.8)
Femur (*n* = 70)	82.1 (66.5–92.5)	96.8 (83.3–99.9)	97.0 (82.2–99.6)	81.1 (68.6–89.4)	25.4 (3.7–175.9)	0.2 (0.1–0.4)	88.6 (78.7–94.9)
Humerus (*n* = 80)	75 (62.1–85.3).0	100 (83.2–100)	100 (n.a.)	57.1 (46.2–67.4)	n.a.	0.3 (0.2–0.4)	81.3 (71.0–89.1)
Overall (*n* = 540)	49.7 (44.0–55.3)	98.2 (95.5–99.5)	97.5 (93.7–99.1)	58.0 (55.3–60.7)	27.6 (10.5–74.0)	0.5 (0.5–0.6)	69.8 (65.8–73.7)

PPV = positive predictive value, NPV = negative predictive value, LR^+^ = positive likelihood ratio, LR^−^ = negative likelihood ratio, n.a. = not applicable, 95% CI = 95% confident interval, XR = plain radiography. *n* refers to the number of “XR before CT imaging” processes in the respective region.

## Supplementary Materials

The following are available online at https://www.mdpi.com/2077-0383/9/1/97/s1, Supplementary Methods: Calculation of injury severity score and estimation of cumulative radiation doses, Supplementary Results: Baseline demographics, way of presentation, trauma mechanism and injury severity, Table S1: Center specific proceedings for emergency imaging (*n* = 4901), Table S2: Total numbers and percentages (%) of accurate and inaccurate diagnosis in “XR before CT examination” imaging processes (*n* = 540), Table S3: Comparison of effective dose estimations (mSv) (median, IQR) per patient (*n* = 2484), Table S4: Summary of effective dose estimations (mSv) (median, IQR) depending on body region (in *n* = 2484 patients) in comparison to previously published data (mean, standard deviation).
