# Supplementary material for: Prevalence of Fractures and Diagnostic Accuracy of Emergency X-ray in Older Adults Sustaining a Low-Energy Fall: A Retrospective Study
Source: J Clin Med. 2019 Dec 30;9(1):97. doi: 10.3390/jcm9010097 (PMC7019509; doi:10.3390/jcm9010097)
Supplement: Supplementary file 1 [file jcm-09-00097-s001.pdf]

Supplementary Materials

Supplementary Methods

Calculation of injury severity score and estimation of cumulative radiation doses

The injury severity score (ISS) (range 1 to 75) has been calculated based upon the abbreviated injury scale (AIS) [1], which has been extracted from the final board certified radiologists' and discharge reports. Taken the highest AIS (range 1 to 6) of the three (AIS_A, AIS_B, AIS_C) most severely injured body regions, the ISS has been determined as: $ISS = AIS_A^2 + AIS_B^2 + AIS_C^2$.

Estimated cumulative radiation doses have been defined as the sum of all effective doses in Millisievert (mSv) resulting from all diagnosis assuring imaging studies including plain radiography (XR) and computed tomography (CT) per case and index visit. Effective doses for every single XR study have been related to the dose area product (DAP), and for CT studies to the dose length product (DLP). DAP and DLP have been retrieved from the individual dose reports (Radiological Information System RIS, Syngo Workflow, Siemens Healthcare, Erlangen, Germany or PACS Syngo Imaging 2010, Siemens Healthcare, Erlangen, Germany). For calculation of effective doses, DAP and DLP have been multiplied with an examination-specific conversion coefficient (for XR: E_{103}/DAP [2], for CT: E_{103}/DLP, for the cervical spine [3] and for every other body regions [4]). Incomplete or missing dose report data sets have been excluded from calculation and statistical analysis.

Supplementary Results

Baseline demographics, way of presentation, trauma mechanism and injury severity

The overall rate of hospital admission is 66.2%, differing between Basel (64%) and Munich (68.4%) ($p = 0.012$, Fisher's exact test), with significantly higher ($p<0.001$, Fisher's exact test) admission rates of patients who have been presented with emergency medical services (EMS), as compared to walk-in patients. Overall in-hospital-mortality is 3.3%, with no differences ($p = 0.3$, Fisher's exact test) between the study centers. In Basel, a significantly higher ($p<0.001$, Fisher's exact test) in-hospital mortality of patients presenting with EMS has been observed. Overall 113/1,879 (6.0%) patients have been admitted to intensive care units. In 2,451/2,839 (86.3%) cases, the trauma mechanism has been a fall from standing height. In 2,544/2,839 (89.6%) cases, presentation has been within 24 hours after the incident. Median injury severity score (ISS) is 3 (range 1–38, IQR 3–4), with no differences between the centers ($p = 0.42$, T-test). Non-injurious falls, with an ISS of 0, have been observed in 377/2,839 (13.3%) cases. Hospital-admission rate of patients with diagnosed fractures is 88.4% (517/585). This was different from patients without fracture diagnosis, of whom 1,362/2,254 (60.4%) have been admitted to the hospital ($p<0.001$, Fisher's exact-test).

Supplementary Tables

Table S1. Center specific proceedings for emergency imaging (n = 4,901).

	**XR only**		**CT only**		**XR before CT**	
	***p*-value**	**OR (95% CI)**	***p*-value**	**OR (95% CI)**	***p*-value**	**OR (95% CI)**
Cervical spine	<0.05	0.37 (0.15–0.89)	<0.001	2.79 (2.39–3.25)	0.46	n.a.
Thoracic spine	<0.001	0.29 (0.19–0.45)	<0.05	1.53 (1.2–1.97)	0.13	n.a.
Lumbar spine	<0.001	0.57 (0.43–0.77)	<0.05	1.36 (1.04–1.78)	0.19	n.a.
Chest	<0.001	0.6 (0.51–0.71)	0.65	n.a.	<0.05	0.45 (0.27–0.75)
Pelvis	<0.05	0.74 (0.61–0.9)	0.69	n.a.	0.36	n.a.
Femur	<0.001	0.65 (0.53–0.78)	0.15	n.a.	0.42	n.a.
Humerus	<0.05	0.67 (0.53–0.85)	<0.05	1.48 (1.95–2.08)	0.59	n.a.

OR > 1 = favors imaging in Munich, OR < 1 favors imaging in Basel. OR = odds ratio, 95% CI = 95% confidence interval, CT = computed tomography, n.a. = not applicable, XR = plain radiography.

Table 2. Total numbers and percentages (%) of accurate and inaccurate diagnosis in "XR before CT examination" imaging processes (n = 540). .

	**XR^-/CT^-**	**XR^+/CT^+**	**XR^+/CT^-**	**XR^+/CT^{++}**	**XR^-/CT^+**
All regions (n = 540)	220 (40%)	157 (30%)	4 (< 1%)	62 (11%)	97 (18%)
Cervical spine (n = 15)	9 (60%)	1 (7%)	0	0	5 (33%)
Thoracic spine (n = 33	13 (39%)	8 (25%)	0	0	12 (36%)
Lumbar spine (n = 76)	31 (41%)	26 (34%)	0	1 (1%)	18 (24%)
Thorax (n = 68)	44 (65%)	5 (7%)	2 (3%)	4 (6%)	13 (19%)
Pelvis (n = 194)	72 (37%)	38 (19%)	1 (< 1%)	48 (25%)	35 (18%)
Femur (n = 70)	30 (43%)	32 (46%)	1 (1%)	1 (1%)	6 (9%)
Humerus (n = 80)	20 (25%)	45 (56%)	0	8 (10%)	7 (9%)
Others (n = 4)	1 (25%)	2 (50%)	0	0	1 (25%)

CT = computed tomography, XR = plain radiography. XR+/CT- = fracture in XR, not confirmed in CT (false positive XR); XR-/CT- = no fractures detected in XR and CT (true negative XR); XR+/CT+ = fracture found in XR and CT (true positive XR); false negative XR: XR+/CT++ = additional fracture(s) in CT and XR-/CT+ = fracture(s) detected solely in CT.

Table S3. Comparison of effective dose estimations (mSv) (median, IQR) per patient (n = 2,484).

	XR and CT (n = 813)	XR before CT (n = 354)	CT only (n = 218)	cCT only (n = 1,099)	p-value
XR	0.02 (0.00–0.02)	0.06 (0.02–0.33)[a]	n.a.	n.a.	<0.001
CT	3.91 (1.42–4.98)	5.29 (3.17–9.27)[a]	9.14 (5.94–15.6)[b]	4.29 (1.46–5.15)	<0.001
Total	4.02 (1.47–5.12)	5.50 (3.17–9.27)[a]	9.14 (5.94–15.6)[b]	4.29 (1.46–5.15)	<0.001

[a] p<0.001 compared to XR and CT; [b] p<0.001 compared to XR and CT/XR before CT/cCT only (FDR correction according to Benjamini-Hochberg). cCT = cerebral computed tomography, CT = computed tomography, IQR = interquartile range, mSv = Millisievert, n.a. = not applicable, XR = plain radiography, XR and CT = XR and CT examinations of independent body regions, XR before CT = XR before CT of the same body region.

Table S4. Summary of effective dose estimations (mSv) (median, IQR) depending on body region (in n = 2,484 patients) in comparison to previously published data (mean, standard deviation) [5].

	**Effective dose XR**	**Effective dose CT**	**Effective dose CT[5]**
Head	n.a.	1.40 (0.17–0.19)	n.a.
Cervical spine	0.09 (0.06–0.22)	3.29 (0.25–1.54)	4.36 ± 2.03
Thoracic spine	0.23 (0.23–0.61)	12.68 (9.06–16.77)	17.99 ± 6.12
Lumbar spine	0.62 (0.39–1.03)	8.08 (4.57–9.67)	19.15 ± 5.63
Chest	0.02 (0.01–0.02)	3.57 (3.52–5.18)	5.27 ± 1.68
Pelvis	0.02 (0.02–0.03)	3.16 (1.54–2.39)	4.85 ± 1.74
Humerus	0.02 (0.03–0.04)	n.a.	2.06 ± 1.52
Femur	0.02 (0.02–0.04)	0.048 (0.02–0.08)	0.16 ± 0.12

CT = computed tomography, IQR = interquartile range, mSv = Millisievert, XR = plain radiography, n.a. = not applicable.

References

1. Gennarelli, T.A.; Wodzin, E.; Association for the Advancement of Automative Medicine. Abbreviated Injury Scale 2005: update 2008. Barrington Ill. 2008.

2. Wall, B.; Haylock, R.; Jansen, J.; Hillier, M.; Hart, D.; Shrimpton, P. *Radiation risks from medical X-ray examinations as a function of the age and sex of the patient*; Health Protection Agency, center for Radiation, Chemical and Environmental Hazards Chilton, Didcot Oxfordshire, 2011.

3. Deak, P.D.; Smal, Y.; Kalender, W.A. Multisection CT protocols: sex-and age-specific conversion factors used to determine effective dose from dose-length product. *Radiol.* **2010,** *257*, 158–166.

4. Shrimpton, P.C.; Jansen, J.T.; Harrison, J.D. Updated estimates of typical effective doses for common CT examinations in the UK following the 2011 national review. *Br. J. Radiol.* **2015,** *89*, 20150346.

5. Biswas, D.; Bible, J.E.; Bohan, M.; Simpson, A.K.; Whang, P.G.; Grauer, J.N. Radiation exposure from musculoskeletal computerized tomographic scans. *J. Bone Joint Surg. Am.* **2009,** *91*, 1882–1889.
